# The Local Treatment of Eczema

**Published:** 1878-11

**Authors:** Henry G. Piffard


### THE LOCAL TREATMENT OF ECZEMA.

By HENRY G. PIFFARD, M.D.

Eczema is the most frequent, one of the most obstinate, and certainly the most important of all the cutaneous affections. Its successful management requires a judicious combination of internal and external treatment, with, in addition, proper hygienic attention. Of these the hygienic is the simplest in its applications, insomuch as a clear conception of the nature of the disease immediately suggests the proper rules of diet, exercise, and the like. The internal treatment—that is, the use of drugs, is the most important, but, at the same time, the most intricate portion of the treatment, and will be considered in its details on another occasion. The local treatment stands midway in importance between the internal and hygienic, and midway also as regards simplicity.

The rule of local treatment is somewhat limited, but if we desire to do our best for the patient, its proper application should not be neglected. In a few cases local treatment alone will succeed in dissipating the lesion, but will not prevent or retard a relapse; in many cases it will materially assist the internal treatment in abridging the duration of the manifestations of the disease, and in a certain number it will modify the subjective phenomena.

Eczema presents many phases varying with the stage, character of the primitive lesion, degree of inflammatory action, individual peculiarity of the patient, complicating circumstances, etc.; but in all of these cases the indications for treatment are so clear that, once rightly appreciated, many of the apparent difficulties disappear.

In no affection with which we are familiar is it so important that the idea of a routine treatment based upon nosology should be abandoned. As regards the internal treatment, it is the *patient*, with all his functional or organic derangements, that demands consideration; in the local treatment it is the cutaneous *lesion* that must be studied and cared for. We must in both cases remember that the conditions actually present in one patient are seldom exactly duplicated in another, and, consequently, that treatment which is best for the first may not, and probably will not, be best for the second. In other words, we must individualize the cases in the strictest manner.

As the present article concerns the lesion only, we will make a brief allusion to the conditions most frequently present, and indicate the principles of treatment that find their application under the varying circumstances of the case.

Every outbreak of eczema commences with a prodromal period of local cutaneous congestion, characterized by heat, redness, slight or almost imperceptible swelling, and certain subjective sensations, which attract attention to the parts. From the appearances alone it will be often difficult to decide what form of cutaneous disease is impending, just as during the first day of an active febrile movement we may be unable to predict the character of the disease that will be developed on the morrow.

This period of congestion is rarely presented to the eye of the physician, except when it occurs in patients who are already suffering from more advanced eczematous lesions in other parts of the body, and who have already come under treatment for them.

Under these circumstances we have known the application of solid nitrate of silver to cause a disappearance of congestions that we supposed would have otherwise developed into frank eczemas.

This prodromal congestion, if uninterfered with, usually eventuates in some one of the so-called special primary lesions of the disease. These are six in number. In the first place, the active congestion may give place to a passive one of indefinite duration, characterized by redness, and often a trace of fine desquamation with possibly a little occasional moisture, alternating with the more usual dryness. The cases were formerly classed as chronic erythemata, but a closer study has convinced most dermatologists that they are essentially eczemata. Little attention has been paid to this form in the text-books, but an admirable delineation of the affection will be found in Dr. Duhring's Atlas. The congestion is usually accompanied with a moderate amount of subjective heat, or itching. This form of eczema is more frequently on the face than elsewhere. The most effective treatment for this variety is internal, but still a great deal of assistance is afforded by external, means employed in conjunction with the latter. The indications are to reduce the congestion, and to relieve the itching. To accomplish the former, the ordinary well-known astringents may be employed. In addition, we have derived benefit from the application of a solution of bromide of potassium in rose-water and glycerine, varying in strength from ten to twenty grains to the ounce. Fluid extract of ergot, rubbed up with cold cream, and a similar preparation of arnica root are also of service. The pruritus, however, must be attended to. This ceases with the congestion, but as this latter will not always subside with wished-for rapidity, antipruritics are often advisable. These may be employed separately or combined with the other applications. Besides the well-known antipruritics, hydrocyanic acid, chloroform, etc., the mixture in equal parts of chloral hydrate and camphor, introduced by McCall Anderson, is worthy of special mention. This mixture, in the proportion of ten to twenty grains to the ounce of ointment, will sometimes greatly palliate the itching.

In the majority of cases, however, instead of the simple chronic congestion, we find a development of certain special lesion, which consist in either vesicles, pustules, papules, fissures, or an exfoliation of the horny layer of the epidermis, or there may be a mixture of two or more of them. This condition is usually termed the first stage, and as regards the vesicles and pustules, lasts for a day or two only. It rarely comes under notice, and requires little in the way of treatment, other than the application of cooling lotions, or better, either the black or yellow wash (mercury and lime-water). To the first stage succeeds the second, characterized by exudation and crusts, specially marked in the vesicular, pustular, exfoliative varieties, less so in the others. The accumulation of secretion and crusts in this stage necessitates ablution, but unfortunately the contact with water proves very irritating in many cases, often causing a decided aggravation of the patient's sufferings and a prolongation of the trouble. If, however, we bear in mind the condition present, namely, the skin deprived of its horny epidermis, but with the delicate and succulent cells of rete Malpighii exposed, we can readily understand why the water proves irritating. It is due to endosmosis, causing tumefaction, and perhaps rupture of the cells. The remedy is equally apparent. It is only necessary to use, instead of water, a fluid whose specific gravity is about the same as the serum of the blood. A mixture that we frequently employ is rose-water, to which is added a little glycerine and chloride of sodium. This will be found much less irritating than pure water.

The crusts being removed, the cleansed parts are in a condition to benefit by some mechanical application, usually in the form of ointment. Of these, the oxide of zinc, when nicely made, is perhaps the best when a protective application alone is needed. It is probably not to any great extent curative, its chief office being to shield the parts from friction and atmospheric influences. The tincture of benzoin it contains, however, probably exerts a soothing influence. The most effectively curative ointments in this stage and condition of eczema, are those containing some preparation of mercury: the ammoniated mercury, the nitrate, and the black oxide. The two first may be employed in ointments of officinal strength, or somewhat diluted, the third in the proportion of ten grains to the ounce. Lead comes next to mercury in usefulness, and is usually employed in the form of *ungt. diachyli.* This, to be of service, must be carefully made, and quite fresh, as it easily becomes rancid and irritating. The "glycerole of the subacetate of lead" (Squire's formula) is not open to this objection. These ointments must be used with caution if the affected surface is extensive, as we have known both mercurial and plumbic symptoms to arise in consequence of their too free employment.

The pruritus, which is usually present and sometimes severe, invites attention. Unfortunately, it is very difficult to relieve. The chloral mixture above referred to should not be applied to a surface deprived of its epithelium, in consequence of the pain it produces, and chloroform should not be used in connection with the lead or mercurial ointments, as it greatly promotes the absorption of these metals. It may, however, be used with the zinc. The ointment containing it must, of course, be kept closely stopped to prevent its evaporation. Decided relief to the itching is sometimes obtained by adding to any of the ointments mentioned a little tincture of Hamamelis Virginica. The best preparation is made from the fresh plant. The various "extracts," "double extracts," "red extracts," fluid extracts, etc., in the market represent but a portion only of the virtues of this plant. Country physicians would do well to make their own tincture of hamamelis, using the bark of the smaller limbs or twigs, and macerating it for a few weeks in sufficient 80 per cent. alcohol to cover it. By this means they can obtain a good tincture very much cheaper than a reliable article can be had in the market. Hamamelis is a drug too highly estimated by the public, but too much neglected by the profession. Stramonium and conium are also useful antipruritics. The white precipitate or black oxide may be added to the *ungt. stramonii*, or tinct. stramonium may be added to the ungt. hydrarg. nit. In spite of these the itching will often prove obstinate, and disappear only on the cure of the eruption itself.

When an acute eczema has passed through the period of exudation and crusting, and enters the third stage, characterized by redness, dryness, and scaling, the changed condition will demand a change of treatment. Here the mercury, zinc, lead, etc., are of comparatively little service, and should be replaced by some preparation of tar. Of these the most important are the *ol. picis*, *ol. rusci*, and *ol. cadini*. The last, when genuine (which is seldom the case), is the best. The tar is mixed with simple ointment in the proportion of one or two drachms to the ounce. A useful preparation belonging to the same category is the "*olio di maiz guasto*," much used in Italy. It is prepared from corn.

Thus far we have spoken of acute eczema only, and more particularly of the vesicular, pustular, and exfoliative forms.

In the fissured form, especially on the palms of the hands and behind the ears, we have found plumbago (the best for this purpose is known as "photographic graphite") in ointment (1–10), or mixed with lycopodium or some other inert powder, exceedingly valuable.

When an eczema becomes chronic, it does so either from sheer indolence or in consequence of excessive infiltration. If the indolence is marked by decided venous stasis, dark bluish red color, etc., the hamamelis before mentioned will be found specifically useful; if, however, this feature is not present, or the color of the patch is rather paler than is usual in eczema, the ham. V. will not be of much, if any use. Under these circumstances we need stimulating, *i. e.*, irritating applications. The basis of these may be hydrarg. biniod., hyd. bichlor., potass. iod., iodine, cantharides, croton oil, and many others that will immediately suggest themselves. The first three may be prescribed in ointment, the last three should be applied by the physician—the iodine in tincture and the cantharides in collodion. The croton oil is very conveniently used in the form of solid cylindrical sticks, made by melting together equal parts of croton oil and white wax, and pouring the mixture into paper molds. A single application of either of these irritants is often sufficient to change an indolent patch of eczema into an active one, which then only requires the treatment appropriate to the second stage of ordinary acute eczema to bring about a cure within a reasonable period.

Quite recently we have obtained excellent results by a process that we believe is original—namely, the hypodermic injection of the arseniate of sodium into the eczematous patch. We use solutions of one-fifth per cent., onehalf per cent., and one per cent. If there be a single patch of moderate size, a single injection of five to ten minims of the one per cent. or one-half per cent. solution is made. If the patch is larger, or if there are several of them, the weaker solutions are employed, and two or more punctures made in the larger patches or distributed among the smaller ones. The injections are to be repeated at intervals of two or three days, *p. r. n.* As yet we have seen neither abscess nor undue reaction. If the physician will take the precaution to obtain pure arsenite of sodium and distilled water, and make the solution himself, he will be more likely to obtain good results than if he leaves the fabrication of the solution to some apothecary's clerk.

A chronic eczema characterized by excessive infiltration rarely exhibits any tendency to heal until the infiltration has in a measure been dissipated. The lead, zinc, and mercurial ointments will rarely prove of much service in these conditions. The special irritant applications just mentioned will do more harm than good, and will probably increase the infiltration. Its removal, however, may frequently be accomplished by the strong alkaline lotions. If *liq. potassæ* or a stronger solution of potash be applied to the infiltrated patch, we will observe, in a few minutes, a more or less copious exudation of clear serum, with, perhaps, a slight temporary increase of swelling. The exudation may continue for some hours, and then gradually diminish. Coincident with the decline of the irritation, the infiltration in part subsides. The application may be renewed at the end of three or four days or a week. The *modus operandi* of the alkaline application is not quite clear. The effects are possibly due to exosmosis, as we have seen the same result follow the application of strong glycerine. Instead of the potash solutions, *sapo viridis*, or ordinary soft-soap, may be used. This should be well rubbed on with a bit of moistened flannel, till the exuding serum has a slight tinge of red; the application to be repeated once or twice a week, if necessary—emollients to be used in the intervals.

We may also attempt the reduction of the infiltration by stimulating the absorptive function of the sanguineous and lymphatic capillaries. The pathological condition present consists in a superabundance of small white cells. Whether these are outwandered leucocytes, or proliferated connective-tissue corpuscles, is a question not yet settled. The present problem is to get them away from the part of the skin in which they have accumulated. Which set of capillaries performs the principal, or perhaps the entire work in this matter, we frankly confess we do not know. Certain it is, however, that "stimulation of the absorbents" may be effected in several ways. The most effective of these is kathodic galvanism. When this is impracticable, we are accustomed to rely upon some of the more active so-called "acro-narcotics" of the indigenous materia medica. Among these hydrastis and its derivatives hold a first rank. Next in usefulness, in our own experience, has been the iris versicolor. This is met with in trade as a tincture made from the fresh plant, as a fluid extract, and as a "concentrated tincture" (Keith's) made from the dried plant. Here, again, the country practitioner has an advantage over his urban brother, insomuch that he can, at small expense, make for himself a good tincture from either the fresh or the freshly-dried root, as he desires. We prefer to rely upon the fresh tincture, as the virtues of the dried root become impaired by long keeping. (*Vide* U. S. Disp.) In using the iris versicolor, from ℨ ss. to ℨ i. are mixed with simple ointment and rubbed up until the alcohol is evaporated. Another tincture that may be usefully employed in the same manner is that of the viola tricolor. This is not strictly an indigenous plant (being naturalized from Europe.) The imported tincture is the one we rely on. That made from the garden plant (cultivated for its flowers) is comparatively worthless. We are not aware that the *v. tricol.* grows wild in any part of this country. The *v. pedata* (*vide* Disp.), however, is found from "New England to Illinois and southward" (Gray). As the active principle of the various violets is believed to be the same, it is possible that the native species, especially the *v. pedata* (*vide* Disp.) may prove as useful as the foreign.

After the infiltration has been in part or wholly removed by some of the means indicated, the patch of eruption will be in a condition to benefit by the mercurial ointments, etc., followed, if necessary, by tarry applications.

The whole of the foregoing relates to eczemas of the general surface. In certain special locations, however, a few modifications of treatment are desirable. When the affection is located upon the scalp in children, and is extensive, the crusting may be very great and the parts become the home of numerous pediculi. Under these circumstances, delphine or kerosene will destroy the insects. Poulticing will soften and aid in removing the crusts, and cutting the hair will greatly facilitate recovery.

When eczema attacks the hairy portions of the face, the morbid action is sometimes propagated to the lining membranes of the hair follicles (outer and inner root-sheaths), constituting one of the affections which commonly pass under the names of mentagra and sycosis. In these cases it is necessary to remove by epilation all the hairs that proceed from the diseased follicles, in order that the remedial application may penetrate them. In fleshy women eczema sometimes succeeds intertrigo of the sub-mammary and genital regions. In these cases dusting powders play an important role.

Eczema of the lower extremities, especially of the legs, is not unfrequently complicated with varicosis and very considerable infiltration. In the former of these conditions, hamamelis, and in both of them elastic compression, will prove of great service.

Lastly, indolent and thickened eczemas of the palms and soles are often exceedingly obstinate. The thickened epidermis may be rubbed down with sand-paper or pumice stone, and the parts enclosed (at night) with some impermeable fabric (rubber gloves, etc.) The cutaneous exhalations thus retained macerate the parts and excite a healthier action.

The successful management of eczematous lesions necessarily demands an exact appreciation of the conditions present, a knowledge of the means by which they may be remedied, and the proper application of the means.—*New York Medical Record.*

